# Synthesizing comprehensive models for health behavior studies: connecting dots and revealing links on health enhancement through a meta-analysis approach

**DOI:** 10.3389/fpsyg.2025.1518834

**Published:** 2025-04-09

**Authors:** Ying Kai Liao, Yo-Yu Liu, Wann-Yih Wu, Hsin-Kuang Chi, Kuo-Chung Huang, Bich-Hang Vuong

**Affiliations:** ^1^Program of International Business, Nanhua University, Chiayi, Taiwan; ^2^Department of Business Administration, Nanhua University, Chiayi, Taiwan

**Keywords:** health behavior, Self-Determination Theory (SDT), Technology Acceptance Model (TAM), Value Adoption Model (VAM), implementation intentions, Health Action Process Approach (HAPA), meta-analysis

## Abstract

**Objective:**

To systematically synthesize the impact of autonomy and key theoretical models, including Self-Determination Theory (SDT), Technology Acceptance Model (TAM), Value Adoption Model (VAM), Theory of Planned Behavior (TPB), and Health Action Process Approach (HAPA), on health behavior and behavior change determinants, focusing on self-determination and intrinsic motivation as central drivers of independent health decisions.

**Methods:**

A meta-analysis was conducted on 135 studies published between 2005 and 2023, with a total sample size of 53,242 participants. We searched the Web of Science and PubMed databases for relevant literature. The theoretical frameworks explored include SDT, TAM, VAM, TPB, and HAPA. Data analysis was performed using Review Manager 5.4.1 software, with effect size calculated as the standardized mean difference with 95% confidence intervals. Heterogeneity was evaluated using I^2^ statistics.

**Results:**

(1) SDT significantly impacted motivation, while TAM and VAM influenced perceived value [SMD = 0.96 > 0.8, 95% CI (0.68, 1.24), *P* < 0.001]. (2) Implementation intentions, action planning, and coping planning were key moderators of health behavior change (I^2^ = 65.4%). (3) HAPA's structured planning strategies positively impacted long-term health behavior changes.

**Conclusion:**

Autonomy, self-determination, and intrinsic motivation play a crucial role in health behavior change. Integrating theoretical models such as SDT, TAM, VAM, and HAPA facilitates structured approaches to health interventions, emphasizing implementation intentions, action planning, and coping strategies.

## 1 Introduction

### 1.1 Research background

The gap between intentions and actual health-related behaviors has been a focal point of research, particularly in behavioral psychology. Social-cognitive theory (Bandura, 1977) posits that intention is the primary predictor of behavior change, yet many individuals act in ways that contradict their health-related intentions. Two main categories of models have emerged to address this issue: continuum models and stage models (Scholz et al., 2008).

Continuum models, namely the Theory of Planned Behavior (Ajzen, 1991) and Protection Motivation Theory (Rogers, 1975), assert that behavior stems directly from intention and is shaped by attitudes, beliefs, and values. While widely used, these models have limitations, primarily in their assumption of a direct, linear relationship between intention and behavior. They fail to account for the “intention-behavior gap”—a significant gap when individuals fail to act on their intentions. These models treat the gap as a “black box,” with little exploration into the factors that disrupt the progression from intention to behavior.

In response to these shortcomings, stage models specifically the Trans theoretical Model (Prochaska et al., 2008) have emerged, emphasizing that behavior change occurs in distinct stages. However, despite their intuitive appeal, stage models like the Trans theoretical Model have been criticized for their arbitrary stage divisions and limited predictive ability in explaining health behavior changes (Lippke et al., 2007). Even within stage models, the gap between intention and behavior must be explained more.

The Health Action Process Approach (HAPA) model (Schwarzer, 2008) represents a significant development in this area by separating the process of health behavior change into two phases: pre-intentional (e.g., contemplation) and post-intentional (e.g., planning and coping). By differentiating between these phases, HAPA offers greater explanatory power in linking intentions with actual behavior, making it a more robust model for understanding health behavior change. Despite this, a fully integrated model that bridges the benefits of both continuum and stage models is still lacking.

In parallel, Self-Determination Theory (SDT) (Deci and Ryan, 1985) offers an understanding of health behaviors through the lens of motivation. According to SDT, autonomy, competence, and relatedness are essential to fostering self-determined motivation. Autonomy enables individuals to make independent decisions regarding their health, while competence provides the necessary knowledge and skills to act on these decisions. Relatedness, or the sense of belonging and social support, reinforces motivation by linking health behaviors to one's community. Collectively, these components foster self-determined motivation, which is crucial for sustaining long-term health behaviors.

Moreover, models namely the Technology Acceptance Model (TAM) (Davis, 1989) and the Value Adoption Model (VAM) (Kim et al., 2007) complement SDT by focusing on how individuals perceive the value of health behaviors. These models suggest that perceived benefits (e.g., usefulness and enjoyment) and sacrifices (e.g., complexity or cost) shape the perceived value of health behaviors. Integrating TAM and VAM with SDT provides a more comprehensive understanding of how motivation and perceived value influence behavior adoption.

By combining the theory of planned behavior with SDT and VAM, the study enhances our understanding of how motivation, attitudes, and perceived value shape intentions to engage in health behaviors. The HAPA model refines this framework by addressing how planning and coping strategies help translate intentions into sustained action. Together, these models offer a comprehensive approach to understanding the factors that bridge the gap between intention and behavior.

### 1.2 Research objectives

1. To identify the influence of SDT on self-determined motivations in promoting health behavior.
2. To investigate the impact of the TAM and VAM on perceived value concerning health behaviors.
3. To examine the relationships between motivation, perceived value, attitudes, and implementation intentions toward health behaviors.
4. To explore the role of action planning and coping strategies in improving and sustaining health behaviors.

By addressing these objectives, this study aims to provide a more integrated framework incorporating SDT, TAM, VAM, and the HAPA model elements to explain how intentions are translated into long-term health behavior changes. Through this comprehensive approach, the study seeks to offer valuable insights into the psychological and behavioral mechanisms that drive health behavior change.

## 2 Literature review and hypotheses development

### 2.1 The influence of self-determination theory (SDT) on health behaviors

Self-determination theory (SDT) (Deci and Ryan, 1985) posits that individuals' motivation is shaped by three fundamental psychological needs: autonomy, competence, and relatedness. These needs must be satisfied for individuals to experience self-determined, intrinsic motivation toward health behaviors. Autonomy refers to the perception of control and personal choice, which enhances motivation when individuals feel empowered to make health-related decisions. Competence represents an individual's ability to manage challenges and accomplish health goals effectively. When individuals can achieve healthy behaviors, their intrinsic motivation is sustained. Relatedness involves meaningful connections with others, which can strengthen health-related motivation by providing emotional support and a sense of belonging.

These three factors are interdependent, creating a reinforcing cycle that drives motivation. Autonomy allows individuals to align health behaviors with personal values, competence ensures that they can successfully engage in these behaviors, and relatedness fosters a supportive environment for continued engagement. This integrated approach aligns with previous research, consistently showing that autonomy, competence, and relatedness positively influence individuals' motivation to adopt and maintain health behaviors (Hollembeak and Amorose, 2005; Chen and Hypnar, 2015; Mallia et al., 2019). This study thus hypothesizes the following:

H1: Autonomy positively impacts self-determined motivation toward health behaviors.
H2: Competence positively impacts self-determined motivation toward health behaviors.
H3: Relatedness positively impacts self-determined motivation toward health behaviors.

### 2.2 The role of the technology acceptance model (TAM) in health behavior adoption

The Technology Acceptance Model (TAM) (Davis, 1989) offers a framework for understanding how individuals adopt technology, and it has been widely applied in health contexts to explain the use of health-related technologies like fitness trackers and mobile health apps. TAM identifies two key factors—perceived usefulness and enjoyment—that shape individuals' perceptions of the value of these technologies. Perceived usefulness refers to the degree to which individuals believe using a particular technology will improve their health outcomes. In contrast, perceived enjoyment reflects the pleasure derived from using the technology.

When individuals perceive health technologies as valuable and enjoyable, they are more likely to view them positively and integrate them into their daily health routines. These two factors are critical in shaping the perceived value of health-related technologies, ultimately increasing their adoption (Jamal and Sharifuddin, 2015; Khasawneh and Haddad, 2020). Based on these insights, this study proposes the following hypotheses:

H4: Perceived usefulness positively impacts individuals' perceived value of health-related technologies.
H5: Perceived enjoyment positively impacts individuals' perceived value of health-related technologies.

### 2.3 The impact of perceived sacrifice on health behavior adoption

The Value Adoption Model (VAM) (Kim et al., 2007) identifies perceived sacrifice, comprising complexity and cost, as significant factors that negatively influence individuals' perceived value of health technologies. Complexity refers to the perceived difficulty of using health-related technologies, which can become a barrier to adoption if users find them too challenging to navigate. Additionally, perceived cost includes financial and non-financial burdens, which can reduce the perceived value of technology if individuals believe the benefits do not justify the expenses.

Balancing these challenges with user-friendly designs and affordable solutions is essential to enhance the perceived value of health-related technologies (Kim et al., 2007; Lin et al., 2012). Thus, this study hypothesizes:

H6: Perceived complexity negatively impacts perceived value toward health-related technologies.
H7: Perceived cost negatively impacts perceived value toward health-related technologies.

### 2.4 Self-determination motivations, attitudes, and perceived value toward health behaviors

Self-determination theory (SDT) suggests that when individuals' needs for autonomy, competence, and relatedness are fulfilled, they are more likely to develop positive attitudes toward health behaviors. According to the theory of planned behavior, attitudes are critical determinants of behavior (Ajzen, 1991), and SDT posits that intrinsic motivations enhance these attitudes, resulting in stronger intentions to engage in healthy behaviors. The Value Adoption Model (VAM) also posits that perceived value influences behavior by assessing benefits and costs. Integrating SDT, TPB, and VAM, this study explores how self-determination motivations influence attitudes and perceived value, leading to greater health behavior adoption (Chan and Hagger, 2012; Carfora et al., 2022).

H8: Self-determination motivations positively influence individuals' attitudes toward health behaviors.
H9: Self-determination motivations positively influence individuals' perceived value of health behaviors.

### 2.5 Interrelationships among attitudes, perceived value, and implementation intentions

The Theory of Planned Behavior (TPB) (Ajzen, 1991) highlights the critical role of attitudes in shaping behavioral intentions, which, when combined with perceived behavioral control and subjective norms, guide the execution of health behaviors. In health contexts, attitudes—rooted in self-determination motivations as per SDT—positively influence the intention to engage in behaviors. The Value Adoption Model (VAM) complements this by proposing that perceived value mediates the relationship between attitudes and intentions. Specifically, when individuals perceive health behaviors as highly valuable, they are more likely to form strong implementation intentions, which increase the likelihood of behavior adoption (Jamal and Sharifuddin, 2015).

This study proposes a model in which self-determined motivations shape positive attitudes, leading to an enhanced perception of value. This perceived value, in turn, strengthens individuals' intentions to implement health behaviors. Empirical evidence supports this pathway, demonstrating the relationships among attitudes, perceived value, and intentions (Salehzadeh and Pool, 2016; Woo and Kim, 2019). Based on this framework, the following hypotheses are proposed:

H10: Attitudes positively influence perceived value toward health behaviors.
H11: Attitudes positively influence implementation intentions toward health behaviors.
H12: Perceived value positively influences implementation intentions toward health behaviors.

### 2.6 The impact of self-determination motivations on implementation intentions

Self-determination theory (SDT) explains how intrinsic motivation, driven by autonomy, competence, and relatedness, influences implementation intentions toward health behaviors. Autonomy fosters the internalization of health goals, competence enhances individuals' confidence in executing behaviors, and relatedness provides the necessary social support for sustained commitment. These three components create a motivational foundation that promotes the formation of strong intentions to implement health behaviors (Scholz et al., 2008; Hagger et al., 2012).

Self-determined motivations encourage individuals to set health goals and provide the psychological resources to develop actionable plans. Studies across different contexts have supported this link between intrinsic motivation and implementation intentions, confirming the critical role of autonomy, competence, and relatedness in shaping behavior (Deci and Ryan, 2008; Joo et al., 2018). Based on this theoretical foundation, the study hypothesizes:

H13: Self-determination motivations positively influence implementation intentions toward health behaviors.

### 2.7 The impact of implementation intentions on action and coping planning in health behaviors

The Health Action Process Approach (HAPA) provides a structured framework for understanding the progression from health intentions to actual behavior. This model's implementation intentions—detailed strategies about when, where, and how to enact behaviors—serve as a bridge between motivation and action. These intentions are vital for translating general goals into concrete behaviors, guiding action, and coping planning.

Action planning refers to creating specific plans for initiating behaviors, specifically scheduling exercise routines or designing meal plans. With clear implementation intentions, individuals can better develop these precise action plans, enhancing their ability to begin health-related activities effectively. Coping planning, on the other hand, involves identifying potential obstacles that may prevent successful behavior execution and devising strategies to overcome them. For instance, an individual aiming to adhere to a healthy diet may anticipate challenges like social gatherings and develop plans, namely bringing healthy snacks, to manage such scenarios.

Research shows that both planning strategies are critical to maintaining health practices. Action planning starts behavior inside the framework provided by implementation intentions while coping planning provides resilience by assisting people in overcoming obstacles (Araujo-Soares et al., 2009; Schwarzer, 2016). In HAPA, action and coping preparation work together to create a synergistic process that ensures intentions become long-lasting health activities. These procedures are essential for creating enduring health behaviors.

Studies by Rebar et al. (2023), Fleig et al. (2013), and Hagger and Luszczynska (2014) consistently show that implementation intentions significantly impact action and coping planning. Coping planning, in particular, enhances the success of action planning by ensuring individuals remain persistent in facing challenges. Thus, we propose the following hypotheses:

H14: Implementation intentions positively impact action planning.
H15: Implementation intentions positively impact coping planning.
H16: Coping planning positively impacts action planning.

### 2.8 The influence of implementation intention, action planning, and coping planning on health behaviors and health improvement

Within the HAPA framework, action planning and coping planning are pivotal strategies for adopting and sustaining health behaviors, ultimately contributing to improved health outcomes. Action planning involves turning general intentions into detailed, actionable steps, specifically specifying exercise routines, locations, and times. This structured planning helps individuals move from motivation to actual behavior change.

Coping planning, on the other hand, equips individuals to anticipate and address potential barriers. By preparing strategies to overcome obstacles namely time constraints, social pressures, or limited resources, coping planning supports individuals in maintaining health behaviors even under challenging circumstances. For instance, an individual who prepares healthy meals in advance to avoid unhealthy food choices at social events is engaging in effective coping planning.

The dynamic interaction between action and coping planning significantly enhances an individual's capacity to initiate and sustain health behaviors. Studies by de Bruijn et al. (2014) and Allan et al. (2013) demonstrate the significant influence of action planning on health behavior adoption, while Scholz et al. (2008) and Pakpour et al. (2011) highlight the essential role of coping planning in overcoming challenges and maintaining behavior.

Additionally, consistent health behaviors, including regular physical activity and adhering to a balanced diet, are directly linked to improvements in overall health and wellbeing. Empirical affirms that consistently implementing health behaviors improves physical and mental health outcomes studies (Webb et al., 2013; Rogowska et al., 2021).

In summary, combining implementation intentions, action planning, and coping planning provides a comprehensive strategy for translating intentions into sustained health behaviors, ultimately leading to significant health improvements. Based on this discussion, we propose the following hypotheses:

H17: Action planning positively impacts individuals' health behavior.
H18: Coping planning positively impacts individuals' health behavior.
H19: Implementation intentions positively impact individuals' health behavior.
H20: Health behaviors positively impact individuals' health improvement.

**Figure 1:**
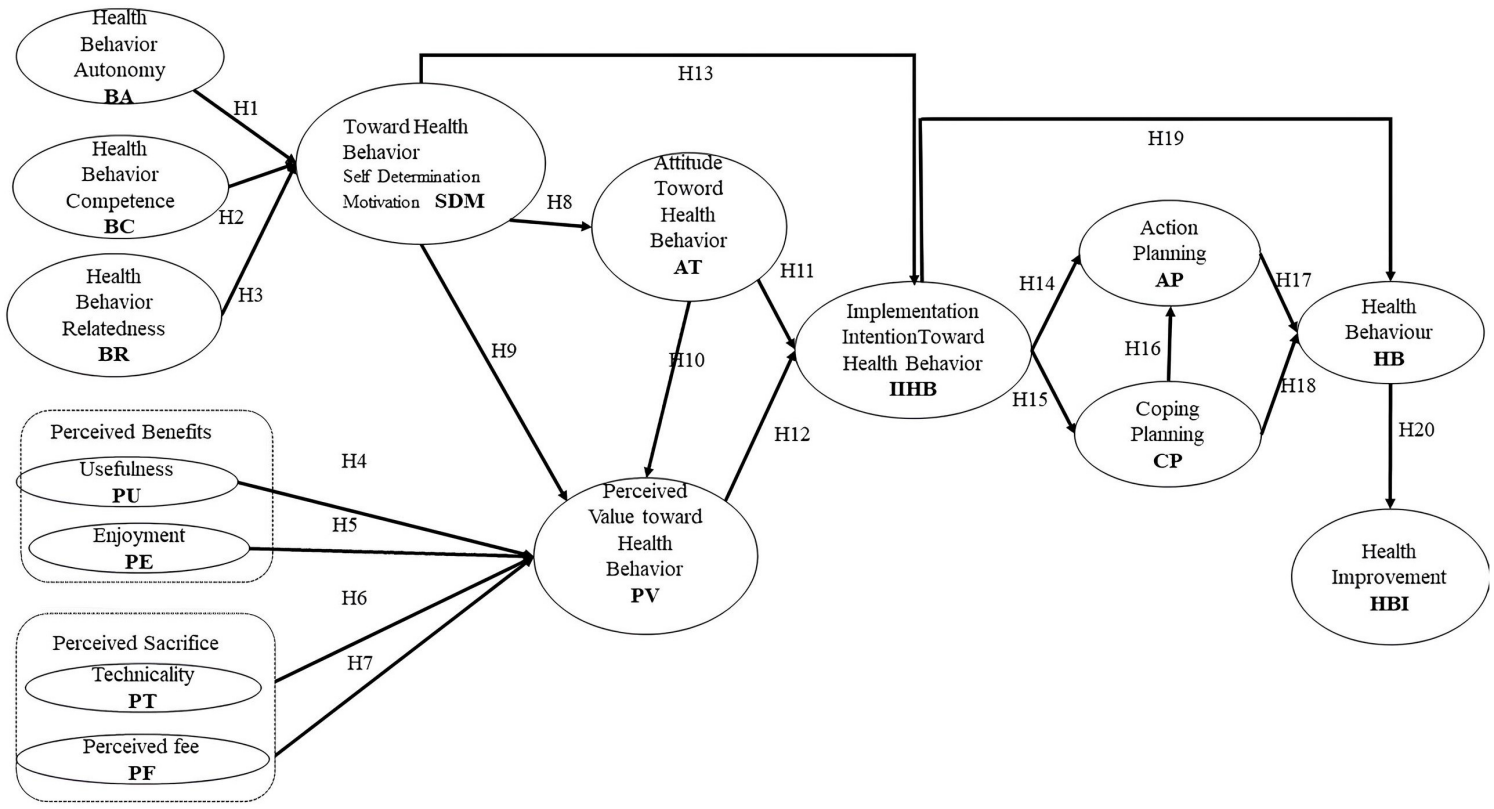
The conceptual framework.

Figure 1 illustrates the research framework derived from the prior discussion and the development of hypotheses. This conceptual framework visually depicts the interaction between key theoretical models—Self-Determination Theory (SDT), Technology Acceptance Model (TAM), Value Adoption Model (VAM), Theory of Planned Behavior (TPB), and Health Action Process Approach (HAPA). It outlines the relationships between various constructs, specifically health behavior autonomy, competence, and relatedness, and their influence on health behavior, motivation, attitudes, planning strategies, and health improvement outcomes.

## 3 Method

This study employed the Comprehensive Meta-Analysis (CMA) software package, which provides a rigorous and systematic approach to synthesizing findings from diverse studies. To ensure the robustness of the meta-analysis, stringent inclusion criteria were applied. Only studies published in peer-reviewed journals, written in English, directly relevant to the research topic, and containing the necessary statistical data were included (Borenstein et al., 2009; Borenstein, 2022). This meticulous selection process was designed to uphold high standards of quality and reliability, thereby enhancing the credibility of the meta-analytic conclusions.

### 3.1 Inclusion criteria

The inclusion criteria were established to comprehensively integrate relevant studies into the meta-analysis: studies must be published in English-language, peer-reviewed journals; directly address the research topic; and report essential statistical measures, namely correlations or standardized regression coefficients, along with sample size information (Borenstein, 2022). The requirements prioritized empirical and quantitative research, strongly emphasized testing hypotheses, and supplied adequate statistical data for analysis.

A thorough search of online databases—including Taylor & Francis Group, Google Scholar, Research Gate, JSTOR, Science Direct, SAGE, and PubMed—was conducted using keywords specifically “Self-Determination Motivation (SDM)”, “Theory of Planned Behavior (TPB)”, “Value Adoption Model (VAM)”, and “Health Action Process (HAPA)”. This extensive search strategy aimed to capture a comprehensive set of relevant studies, ensuring methodological rigor and reliability in the meta-analysis. We focused on studies published between 2005 and 2023, ensuring the most up-to-date research was included. This strategy aimed to provide a solid foundation for meaningful synthesis and significant insights into the research questions by adhering to these criteria.

### 3.2 Meta-analytic strategy

This study employed a comprehensive meta-analysis approach, known for its robustness in synthesizing findings from multiple disciplines (Hunter, 1994; Borenstein et al., 2009). The CMA software was chosen for its intuitive interface and ability to handle various effect size measures, particularly in addressing issues like heterogeneity and publication bias. The program's statistically solid capabilities ensured a thorough and methodical analysis (Aertsens et al., 2009; Borenstein et al., 2009).

### 3.3 Analytical techniques

The study primarily used the correlation coefficient (r) as the effect size measure, with standardized regression coefficients (β) converted to r for uniformity. Statistical significance was determined by calculating 95% confidence intervals (CI) and assessing significance using the Hartung and Knapp (2001) method. The analysis also employed Q-statistics to test the homogeneity of effect sizes. A significant Q-statistic indicated variance heterogeneity, prompting the inclusion of moderators to explain the variability in effect sizes.

Additionally, the Fail-Safe N analysis was applied to evaluate the stability of results, specifying the number of additional studies required to negate the observed effect. The Fail-Safe N's substantial Z value (p < 0.05) strengthened the reliability of the meta-analysis results, even in the presence of non-significant studies (Long, 2001a,b; Orwin, 1983). Fail-Safe N ensures a thorough examination of publication bias and enhances the reliability of the meta-analytic conclusions.

### 3.4 Variable coding

The variable coding procedure in this meta-analysis was rigorously structured to ensure consistency and reliability. Each study was systematically coded for critical variables specifically study characteristics, participant demographics, and relevant outcome measures. To ensure precision, we employed pre-defined coding protocols; unlike prior studies that often produced multiple effect sizes for a single construct, our approach aimed to derive a single, comprehensive effect size per variable for clarity. The research team, which included the authors and a PhD student proficient in the field, independently coded the studies. Pre-coding assessments were conducted to ensure alignment, allowing for methodological consistency and resolving discrepancies. This meticulous process contributed to enhanced reliability in the integration of effect sizes and overall findings of the meta-analysis. Table 1 summarizes data collection for health behavior from the device sources.

**Table 1:** The Table shows the studies included in the meta-analysis.

**Studies alphabetically by source and codes for hypot hesis tests^a^^,^^b^**	
Al-Jubari (2019), 44, (AT-IIHB) Allan et al. (2013), 1, (IIHB-AP, AP-HB, IIHB-HB) Araujo-Soares et al. (2009), 17, (IIHB-AP, IIHB-CP, CP-AP) Bavaresco et al. (2020),4, (AT-IIHB) Brouwer et al. (2010), 37, (AT-IIHB) Cao et al. (2022),45, (PU-PV, PE-PV) Carfora et al. (2022), 40, (SDM-AT) Caudroit et al. (2014), 42, (IIHB-AP, IIHB-CP, CP-AP) Chan and Hagger (2012), 30, (SDM-AT AT-IIHB) Chan et al. (2020),41, (SDM-AT) Chatzisarantis and Biddle (1998), 11, (AT-IIHB) Chen and Hypnar (2015), 24, (BA-SDM, BC-SDM, BR-SDM, SDM-AT) Cheng and Lu (2013), 3, (PV-IIHB) Huang et al. (2014), 19, (PV-IIHB, SDM-IIHB, SDM-PV) Chung et al. (2017),4, (SDM-AT, AT-IIHB) de Bruijn et al. (2012), 32, (AT-IIHB, IIHB-AP) de Bruijn et al. (2014), 18, (AT-IIHB, IIHB-AP, AP-HB, IIHB-HB) Dumitrescu et al. (2011), 29, (HB-HBI) Faes (2011), 11, (AT-IIHB) Fleig et al. (2013), 41, (IIHB-AP) Godinho et al. (2014), 8, (IIHB-AP, IIHB-CP, CP-AP) Grønhøj et al. (2013), 16, (AT-IIHB) Hagger et al. (2012), 5, (SDM-AT, AT-IIHB, SDM-IIHB, IIHB-HB) Hasan and Suciarto (2020), 32, (PV-IIHB, AT-PV) Hollembeak and Amorose (2005), 26, (BA-SDM, BC-SDM, BR-SDM) Hsieh et al. (2019), 50, (IIHB-AP, IIHB-CP, CP-AP) Inoue et al. (2015), 26, (SDM-AT) Jamal and Sharifuddin (2015), 27, (PV-IIHB, PU-PV) Johnson et al. (2016), 48, (SDM-PV) Joo et al. (2018), 8, (SDM-IIHB) Jun et al. (2014), 21, (AT-IIHB) Khasawneh and Haddad (2020), 20, (PU-PV, PE-PV)	Kim et al. (2007), 9, (PU-PV, PE-PV, PT-PV, PF-PV, PV-IIHB) Lee et al. (2012), 36, (PV-IIHB) Lena et al. (2019), 2, (CP-HB) Lin et al. (2012), 9, (PT-PV, PF-PV, PV-IIHB) Lin et al. (2020), 38, (PF-PV, PV-IIHB) Lippke et al. (2004) 35, (IIHB-AP) Liu et al. (2015) 23, (PF-PV, PV-IIHB) Luszczynska et al. (2010), 22, (AP-HB, IIHB-HB) Mallia et al. (2019), 25, (BA-SDM, BC-SDM, BR-SDM) Nystrand and Olsen (2020), 13, (AT-IIHB) Otaibi (2018), 12, (AT-IIHB) Pakpour et al. (2011), 41, (IIHB-AP, IIHB-CP, CP-AP, AP-HB, CP-HB, IIHB-HB) Pena-Garcia et al. (2020), 15, (AT-IIHB) Pfeffer and Strobach (2020),33, (IIHB-AP) Rebar et al. (2023), 42, (IIHB-AP) Rogowska et al. (2021), 43, (HB-HBI) Salehzadeh and Pool (2016), 31, (PV-IIHB, AT-PV) Sniehotta et al. (2005), 11, (IIHB-AP, IIHB-CP, CP-AP) Sørebø et al. (2009), 8, (BA-SDM, BC-SDM, BR-SDM) Souza et al. (2020), 32, (AT-IIHB, PV-IIHB, SDM-IIHB, IIHB-AP, CP-HB) Swaim et al. (2014), 34, (AT-IIHB) Thompson et al. (1994), 6, (AT-IIHB) Vierling et al. (2007), 41, (BA-SDM, BC-SDM, BR-SDM) Webb et al. (2013), 28, (HB-HBI) Willmott et al. (2021), 4, (SDM-AT) Woo and Kim (2019), 6, (AT-IIHB, PV-IIHB, AT-PV) Yu et al. (2019), 46, (PU-PV, PE-PV, PV-IIHB) Yu et al. (2019), 47, (PU-PV, PE-PV, PT-PV, PF-PV, PV-IIHB) Yue and Lu (2022), 14, (SDM-PV) Zehir et al. (2014), 39, (PV-IIHB) Zhu et al. (2022), 14, (IIHB-AP, IIHB-CP, CP-AP, AP-HB, IIHB-HB)

BA, Health Behavior Autonomy; BC, Health Behavior Competence; BR, Health Behavior Relatedness; SDM, Self-Determination Motivation; PU, Perceived Benefits Usefulness; PE, Perceived Benefits Enjoyment; PT, Perceived Sacrifice Technicality; PF, Perceived Sacrifice Perceived fee; AT, Attitude Toward Health Behavior; PV, Perceived Value toward Health Behavior; IIHB, Implementation Intention Toward Health Behavior; AP, Action Planning; CP, Coping Planning; HB, Health Behavior; HBI, Health Behavior Improvement.

a Codes in parentheses.

b Journals are footnoted in alphabetical order.

(1) Annals of Behavioral Medicine (2) AIMS Medical Science (3) Asia Pacific Journal of Tourism Research (4) BMC Public Health (5) British Journal of Health Psychology (6) British Food Journal (7) Building and Environment (8) Computers & Education (9) Decision Support Systems (11) European Journal of Social Psychology (12) Food and Nutrition Sciences (13) Food Quality and Preference (14) Frontiers in Psychology (15) Heliyon (16) Health Education (17) Health Education Research (18) International Journal of Behavioral Medicine (19) International Journal of Culture, Tourism and Hospitality Research (20) International Journal of Electronic Marketing and Retailing (21) International Journal of Hospitality Management (22) International Journal of Clinical and Health Psychology (23) Internet Research (24) Journal of Teaching in Physical Education (25) Journal of Applied Social Psychology (26) Journal of Applied Sport Psychology (27) Journal of Business Research (28)Journal of Environmental Psychology (29) Journal of Oral Science (30) Journal of Science and Medicine in Sport (31) Journal of International Consumer Marketing (32) Journal of Management and Business Environment (33) Journal of Behavioral Medicine (34) Journal of Business Ethics (35) Journal of Sport and Exercise Psychology (36) New Media & Society (37) Nutrition & Food Science (38) Online Information Review (39) Procedia-Social and Behavioral Sciences (40) Public Health Nutrition (41) Psychology of Sport and Exercise (42) Psychology & Health (43) Psychology Research And Behavior Managemen t (44) SAGE Open (45) Telematics and Informatics (46) Total Quality Management & Business Excellence (47) The Journal of Continuing Higher Education (48) Traffic Psychology and Behaviour.

**Figure 2:**
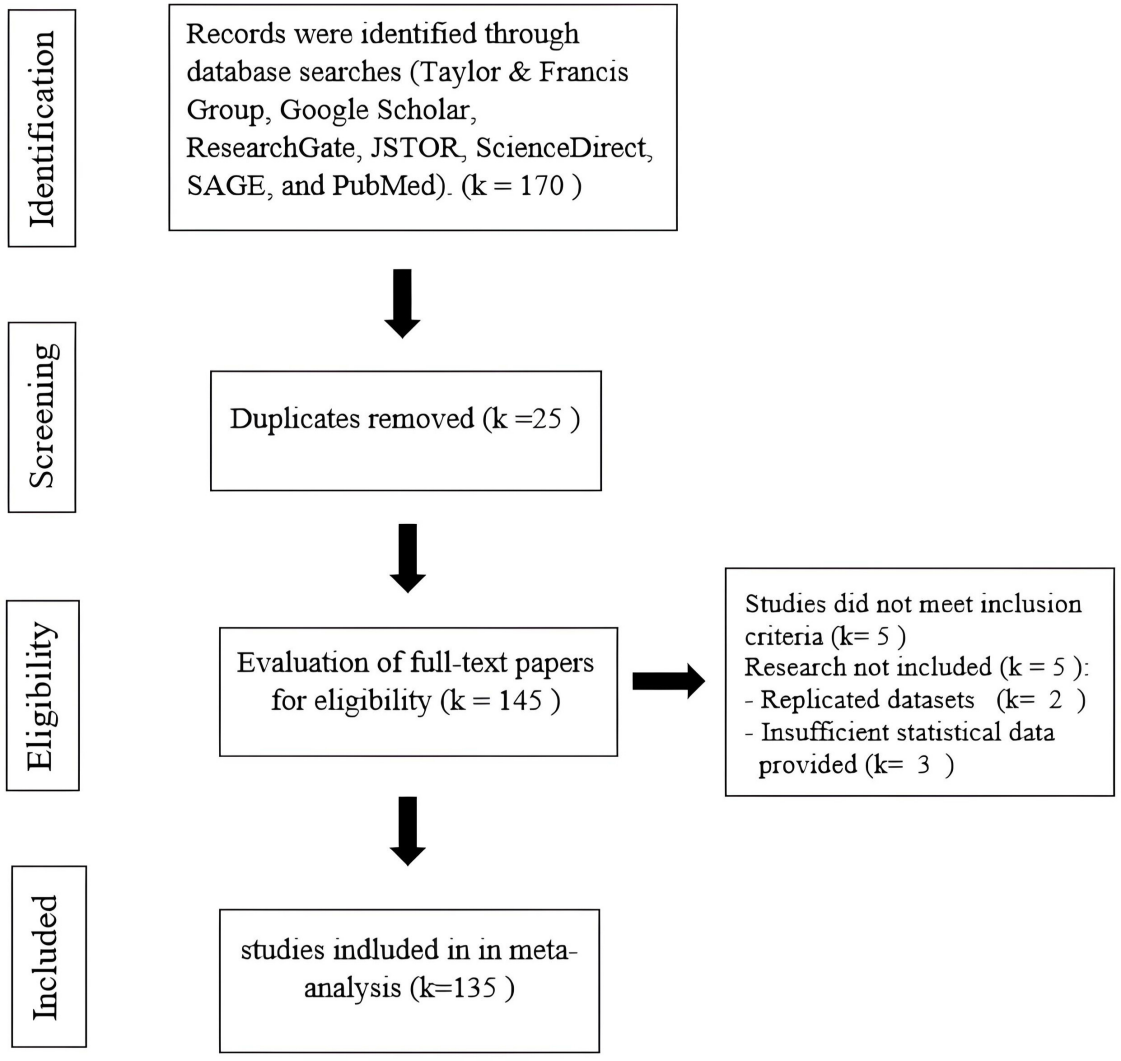
The flow of information through the phases of the review.

We systematically synthesized data from a broad range of empirical studies using meta-analysis. Effect sizes for each hypothesis were aggregated from 170 primary studies, encompassing a total sample size of 53,242. Access to full publications and the AISCAR database was essential, as much of the required statistical data was unavailable in the published articles. Additionally, 35 studies were excluded due to overlapping datasets, leaving 135 studies in the final analysis, as illustrated in Figure 2.

## 4 Results

Table 2 presents the meta-analysis results on health behavior factors, evaluating 20 hypotheses that explore intricate relationships in this domain. Each hypothesis details the specific connections between independent and dependent variables. The results comprehensively summarize effect sizes, confidence intervals, statistical significance, and heterogeneity statistics for each hypothesis. Additionally, the analysis incorporates Fail-Safe N results to offer deeper insights into the interactions among health behavior factors. This thorough examination contributes valuable nuance to the existing body of health behavior research.

**Table 2:** Meta-analysis results of main effects.

**Effect size and 95% confidence**													
**Variable**			***n***	***k***	**Interval**			**Heterogeneity**				**Fail-Safe** ***N***	
**Hyp**.	**Independent**	**Dependent**			**r**	**LCI**	**UCI**	***p*** **-value**	χ^2^	**Q**	**I** ^2^	***Z*** **value**	**Sign**.
H1	BA	SD	2,362	5	0.328	0.165	0.474	0.000	9.488	66.315	93.968	14.23	0.000
H2	BC	SD	2,362	5	0.364	0.087	0.589	0.011	9.488	190.18	97.897	15.49	0.000
H3	BR	SD	2,362	5	0.273	0.063	0.471	0.012	9.488	103.71	96.143	10.82	0.000
H4	PU	PV	2,049	6	0.591	0.392	0.737	0.000	11.07	174.88	97.141	31.08	0.000
H5	PE	PV	1,599	5	0.645	0.521	0.743	0.000	9.488	41.241	90.301	25.98	0.000
H6	PT	PV	683	3	−0.174	−0.246	0.101	0.000	5.991	79.632	97.488	−3.76	0.000
H7	PF	PV	1,250	5	−0.166	−0.219	0.112	0.000	9.488	224.201	98.216	−7.6	0.000
H8	SDM	AT	11,285	9	0.37	0.167	0.544	0.001	15.507	984.88	99.18	37.01	0.000
H9	SDM	PV	1,283	3	0.588	0.099	0.848	0.021	5.991	184.13	98.91	21.18	0.000
H10	AT	PV	953	3	0.579	0.403	0.713	0.000	5.991	25.667	92.208	20.02	0.000
H11	AT	IHB	8,226	21	0.559	0.475	0.632	0.000	31.41	536.15	96.27	54.15	0.000
H12	PV	IHB	4,868	15	0.67	0.655	0.686	0.000	23.685	214.70	93.479	55.38	0.000
H13	SDM	IHB	1,436	4	0.447	0.231	0.621	0.000	7.815	60.569	95.047	18.13	0.000
H14	IHB	AP	4,152	15	0.397	0.371	0.422	0.000	23.685	188.72	92.582	25.16	0.000
H15	IHB	CP	1,488	7	0.439	0.331	0.536	0.000	12.592	35.168	82.939	17.94	0.000
H16	CP	AP	1,488	7	0.49	0.374	0.591	0.000	12.592	44.404	86.48	20.67	0.000
H17	AP	HB	1,702	5	0.356	0.201	0.493	0.000	9.488	46.184	91.33	15.78	0.000
H18	CP	HB	647	3	0.353	0.284	0.419	0.000	5.991	6.102	67.225	9.18	0.000
H19	IHB	HB	2,354	6	0.166	0.126	0.205	0.000	11.07	698.51	99.284	10.51	0.000
H20	HB	HBI	693	3	0.528	0.472	0.579	0.000	5.991	103.365	98.065	13.48	0.000

a Codes in parentheses.

BA, Health Behavior Autonomy; BC, Health Behavior Competence; BR, Health Behavior Relatedness; SDM, Self-Determination Motivation; PU, Perceived Benefits Usefulness; PE, Perceived Benefits Enjoyment; PT, Perceived Sacrifice Technicality; PF, Perceived Sacrifice Perceived fee; AT, Attitude Toward Health Behavior; PV, Perceived Value toward Health Behavior; IIHB, Implementation Intention Toward Health Behavior; AP, Action Planning; CP, Coping Planning; HB, Health Behavior; HBI, Health Behavior Improvement.

### 4.1 Exploring the moderator and principal effects for hypotheses 1, 2, and 3: the intersection of health behavior and self-determination motivation (SDM)

This research examines the fundamental linkages among essential dimensions in health behavior—namely autonomy (BA), competence (BC), and relatedness (BR)—and their association with Self-Determination Motivation (SDM) using an extensive meta-analysis.

Hypothesis H1 investigated the correlation between Autonomy (BA) and Self-Determination Motivation (SDM) using data from 2,362 individuals across five investigations. The meta-analysis indicated a moderate positive impact size (r = 0.328), with a 95% confidence interval (CI) from 0.165 to 0.474 and a highly significant p-value of 0.000. The heterogeneity statistics revealed considerable diversity across the studies (χ^2^ = 9.488, Q = 66.315, I^2^ = 93.968%). The Fail-Safe N analysis, with a Z value of 14.23, underscored the robustness and significance (*p* = 0.000) of the positive association between Autonomy (BA) and SDM.

Hypothesis H2 examined the correlation between Competence (BC) and Shared Decision-Making (SDM). The study, including 2362 people across five investigations, revealed a significant positive impact size (*r* = 0.364) with a 95% confidence interval ranging from 0.087 to 0.589 and a p-value of 0.011. Heterogeneity was substantial (χ^2^ = 9.488, Q = 190.18, I^2^ = 97.897%), indicating diversity across studies. The Fail-Safe N analysis, yielding a Z value of 15.49, validated the robustness of the effect (*p* = 0.000), indicating a significant and dependable link between Competence (BC) and SDM.

Hypothesis H3 investigated the relationship between Relatedness (BR) and SDM. The aggregate sample size was 2362 over five investigations, yielding an effect size of *r* = 0.273, with a 95% confidence interval ranging from 0.063 to 0.471 and a statistically significant *p*-value of 0.012. Despite significant heterogeneity (χ^2^ = 9.488, Q = 103.71, I^2^ = 96.143%), the Fail-Safe N analysis, yielding a Z value of 10.82, confirming the robustness of the results (*p* = 0.000). Notwithstanding the substantial diversity across research, these findings highlight the significant positive correlation between Relatedness (BR) and SDM.

The meta-analysis results underscore the strong and statistically relevant correlations among Autonomy (BA), Competence (BC), Relatedness (BR), and Self-Determination Motivation (SDM). Each construct enhances comprehension of health behavior and motivation, underscoring the significance of these interactions within the framework of health treatments and self-determination theory.

### 4.2 Exploring the interconnected dimensions of Perceived Value (PV): Benefits Usefulness (PU), Perceived Benefits Enjoyment (PE), Perceived Sacrifice Technicality (PT), and Perceived Sacrifice Perceived Fee (PF)

The meta-analysis investigated Hypothesis H4, the relationship between Benefits Usefulness (PU) and Perceived Value (PV), analyzing six studies with a total sample size of 2,049 participants. The analysis revealed a substantial effect size (r = 0.591, 95% CI: 0.392 to 0.737, *p* = 0.000). The statistical tests indicated significant heterogeneity (χ^2^ = 11.07, Q = 174.88, I^2^ = 97.141%), emphasizing the need to consider variations across studies. The Fail-Safe N analysis, with a Z value of 31.08, confirmed the robustness of the observed effect, supporting its statistical significance (*p* = 0.000). This finding underscores a robust positive correlation between Benefits Usefulness (PU) and Perceived Value (PV), offering valuable insights into the interconnected dimensions of perceived value. Hypothesis H5, which explored the relationship between Perceived Benefits Enjoyment (PE) and Perceived Value (PV), was examined through a meta-analysis of 5 studies with a total sample size of 1,599 participants. The analysis indicated a significant positive effect size (r = 0.645, 95% CI: 0.521 to 0.743, *p* = 0.000). Heterogeneity statistics (χ^2^ = 9.488, Q = 41.241, I^2^ = 90.301%) indicated notable diversity across the studies. The Fail-Safe N analysis, yielding a Z value of 25.98, confirmed the robustness and significance of the observed effect (*p* = 0.000). This result highlights the positive correlation between Perceived Benefits Enjoyment (PE) and Perceived Value (PV). Additionally, Hypothesis H6 tested the relationship between Perceived Sacrifice Technicality (PT) and Perceived Value (PV). The meta-analysis included three studies with a sample size of 683 participants. The effect size was negative (r = −0.174, 95% CI: −0.246 to −0.101, *p* = 0.000), indicating a statistically significant negative correlation. Heterogeneity statistics (χ^2^ = 5.991, Q = 79.632, I^2^ = 97.488%) showed substantial variability across studies. The Fail-Safe N analysis, with a Z value of −3.76, confirmed the robustness of the observed effect (*p* = 0.000), highlighting the significant negative correlation between Perceived Sacrifice Technicality (PT) and Perceived Value (PV).

Finally, Hypothesis H7 focused on the relationship between Perceived Sacrifice, Perceived Fee (PF), and Perceived Value (PV), including 5 studies with a sample size of 1,250 participants. The analysis found a significant negative effect size (r = −0.166, 95% CI: −0.219 to −0.112, *p* = 0.000). The heterogeneity statistics (χ^2^ = 9.488, Q = 224.201, I^2^ = 98.216%) highlighted notable variability across studies. The Fail-Safe N analysis, with a Z value of −7.6, confirmed the robustness of the observed effect (*p* = 0.000), underscoring the significant negative correlation between Perceived Sacrifice, Perceived Fee (PF), and Perceived Value (PV).

### 4.3 Empowering health engagement: the dual impact of self-determination motivations on attitudes and perceived Value in health behaviors

The objective of Hypothesis H8 was to examine the influence of Self-Determination Motivation (SDM) on Attitude Toward Health Behavior (AT). Based on 11,285 participants across nine studies, the meta-analysis demonstrated a moderate and positive effect size of r = 0.37 (95% CI: 0.167, 0.544, p = 0.001). Despite this strong positive relationship, significant heterogeneity was detected (χ^2^ = 15.507, Q = 984.88, I^2^ = 99.18%), suggesting notable variability across studies. The Fail-Safe N analysis, with a Z-value of 37.01, reinforced the robustness of the observed effect, emphasizing the crucial role of SDM in shaping favorable attitudes toward health behaviors (*p* = 0.001).

Hypothesis H9 investigated the impact of SDM on Perceived Value (PV) in health behaviors. The meta-analysis, which included 1,283 participants from three studies, found a strong positive correlation with an effect size of r = 0.588 (95% CI: 0.099, 0.848, *p* = 0.021). As with H8, significant heterogeneity was observed (χ^2^ = 5.991, Q = 184.13, I^2^ = 98.91%), indicating considerable variability across the included studies. The Fail-Safe N analysis, with a Z-value of 21.18, further supported the effect's significance, demonstrating that many non-significant studies would negate the observed relationship (*p* = 0.021). This highlights the vital influence of SDM on individuals' perception of value concerning health behaviors.

### 4.4 Holistic health motivation: unveiling the interplay of attitudes, perceived value, and implementation intentions in shaping health behavior choices

The study investigated the influence of Attitudes (AT) on Perceived Value (PV) in health practices under Hypothesis H10. The meta-analysis, which included 953 participants from three studies, found a statistically significant positive effect size of r = 0.579 (95% CI: 0.403, 0.713, *p* = 0.000). Despite this robust positive effect, considerable heterogeneity was observed (χ^2^ = 5.991, Q = 25.667, I^2^ = 92.208%), suggesting substantial variability across the studies. The Fail-Safe N analysis, with a Z-score of 20.02, demonstrated that a significant number of non-significant studies would be required to counteract the observed impact, confirming its strength (*p* = 0.000). These findings emphasize the crucial role that attitudes play in shaping individuals' perceptions of the value of health-related activities.

Further, Hypothesis H11 examined the relationship between Attitudes (AT) and Implementation Intentions toward Health Behavior (IHB). Based on 8,226 participants across 21 studies, the meta-analysis identified a significant positive effect size of r = 0.559 (95% CI: 0.475, 0.632, p = 0.000). However, considerable heterogeneity was detected (χ^2^ = 31.41, Q = 536.15, I^2^ = 96.27%), highlighting variability among the studies. The Fail-Safe N analysis, with a Z-score of 54.15, confirmed the robustness of the observed effect, indicating that many non-significant studies would be required to negate the results. This significant finding (*p* = 0.000) underscores the influential role of attitudes in motivating individuals' intentions to adopt health behaviors.

Hypothesis H12 examined the influence of Perceived Value (PV) on Implementation Intentions regarding Health Behavior (IHB). The meta-analysis, including 4,868 people from 15 trials, demonstrated a significant positive impact size of r = 0.67 (95% CI: 0.655, 0.686, p = 0.000). Substantial heterogeneity was observed (χ^2^ = 23.685, Q = 214.70, I^2^ = 93.479%), indicating diversity across the studies. The Fail-Safe N analysis, yielding a Z-score of 55.38, substantiated the importance of the observed impact, necessitating several non-significant investigations to invalidate the finding. The statistically significant results (*p* = 0.000) highlight the essential role of perceived value in motivating people's intentions to participate in health-related actions.

### 4.5 Exposing the significance of self-determination motivations in promoting health choice empowerment

Hypothesis H13 investigated the impact of Self-Determination Motivations (SDM) on the Intention to Adopt Health Behaviors (IIHB). A statistically significant positive impact size of r = 0.447 (95% CI: 0.231, 0.621, *p* = 0.000) was found in the meta-analysis, which comprised 1,436 participants from 4 investigations. The heterogeneity statistics showed significant variety among the studies with a χ^2^ value of 7.815, a Q-statistic of 60.569, and an I^2^ value of 95.047 percent. This conclusion is further confirmed by the Z-score of 18.13 obtained from the Fail-Safe N analysis, which showed that many non-significant studies would be required to offset the observed effect. This statistically significant result highlights the importance of self-determination motives in encouraging people to choose healthy behaviors (*p* = 0.000).

### 4.6 Facilitating the execution of health behaviors: exploring the interplay among implementation intentions, action planning, and coping planning in the context of strategic health empowerment

The effect of Implementation Intentions toward Health Behavior (IIHB) on Action Planning was the subject of Hypothesis H14. A significant positive effect size of r = 0.397 (95% CI: 0.371, 0.422, *p* = 0.000) was discovered in the meta-analysis, demonstrating a high correlation between the preparation of action plans for health behaviors and implementation intentions. The acquired statistics on heterogeneity (I^2^ of 92.582 percent, Q-statistic of 188.72, and χ^2^ value of 23.685) demonstrate high variability among the included research. With a Z-score of 25.16, the Fail-Safe N value suggests that a sizable number of non-significant studies would be required to offset the observed effect. The result is statistically significant (*p* = 0.000), underscoring the crucial role that implementation intentions play in motivating action planning to encourage the adoption of healthy behaviors. Hypothesis H15 studied the connection between Implementation Intentions toward Health Behavior (IIHB) and Coping Planning (CP). The meta-analysis found a significant positive impact size of r = 0.439 (95% CI: 0.331, 0.536, *p* = 0.000). The heterogeneity statistics—χ^2^ value of 12.592, Q-statistic of 35.168, and I^2^ of 82.939%—indicated high variation among the included studies. The Fail-Safe N value, with a Z-score of 17.94, suggests that a sizable number of non-significant studies would be necessary to invalidate the observed impact. This substantial finding (*p* = 0.000) underlines the importance of implementation intentions in motivating coping planning, which is critical for effectively adopting health habits.

Finally, Hypothesis H16 studied the association between Coping Planning (CP) and Action Planning (AP). The meta-analysis found a positive impact size of r = 0.49 (95% CI: 0.374, 0.591, *p* = 0.000), suggesting a substantial link between coping planning and action planning in health behavior implementation. The heterogeneity statistics—χ^2^ value of 12.592, Q-statistic of 44.404, and an I^2^ value of 86.48%—indicated high variation among the included studies. The Fail-Safe N value, with a Z-score of 20.67, suggests that many non-significant studies would be necessary to offset this effect. The statistically significant finding (*p* = 0.000) underscores the crucial significance of coping planning in generating effective action plans for the successful execution of health behaviors. These findings have practical implications for health behavior empowerment, providing a roadmap for practitioners to develop effective strategies for their clients.

### 4.7 Integrating plans for better health: exploring the linkages between action planning, coping planning, implementation intentions, and health behavior for comprehensive health enhancement

Hypothesis H17 investigated the effect of Action Planning (AP) on Health Behavior (HB). The meta-analysis indicated a considerable positive impact of r = 0.356 (95% CI: 0.201, 0.493, p = 0.000), demonstrating a robust link between action planning and individuals' health habits. The heterogeneity statistics—χ^2^ value of 9.488, Q-statistic of 46.184, and an I^2^ of 91.33 percent —indicate significant variability among the included research. The Fail-Safe N value, with a Z-score of 15.78, shows that many non-significant studies would be needed to counteract this effect. The significant outcome (*p* = 0.000) highlights the critical role of action planning in creating health habits, thereby achieving overall health promotion. Furthermore, Hypothesis H18 studied the association between Coping Planning (CP) and Health Behavior (HB). The meta-analysis found a positive impact size of r = 0.353 (95 percent CI: 0.284, 0.419, *p* = 0.000). The heterogeneity statistics χ^2^ value of 5.991, Q-statistic of 6.102, and I^2^ of 67.225 percent suggest high variability among the studies. The Fail-Safe N value, with a Z-score of 9.18, demonstrates that a substantial number of non-significant studies would be necessary to offset the observed effect. This large finding (*p* = 0.000) highlights the role of coping planning in modifying health behaviors for overall health improvement.

Hypothesis H19 explored how Implementation Intentions toward Health Behavior (IHB) affect actual health behaviors (HB). The meta-analysis identified a robust positive impact size of r = 0.166 (95% CI: 0.126, 0.205, *p* < 0.001). The heterogeneity statistics χ^2^ value of 11.07, Q-statistic of 698.51, and I^2^ of 99.284 percent suggest considerable diversity among the studies. The Fail-Safe N value, with a Z-score of 10.51, shows that a substantial number of non-significant studies would be necessary to cancel the observed effect. This result (*p* = 0.000) emphasizes the enormous impact of implementation intentions on changing individuals' health behaviors, contributing to overall health improvement.

Lastly, Hypothesis H20 investigated the association between Implementation Intentions toward Health Behavior (IHB) and Improvements in Health Behavior (HBI). The meta-analysis indicated a substantial positive impact size of r = 0.528 (95% CI: 0.472, 0.579, *p* = 0.000). The heterogeneity statistics—χ^2^ value of 5.991, Q-statistic of 103.365, and I^2^ of 98.065 percent —suggest considerable diversity throughout the investigations. The Fail-Safe N value, with a Z-score of 13.48, indicates that many non-significant studies would be necessary to negate the influence. This statistically significant outcome (*p* = 0.000) underscores the vital role of implementation goals in attaining enduring health behavior modifications for comprehensive health promotion.

## 5 Discussion

This study aimed to develop a comprehensive framework to explain individuals' health behaviors and pathways to health improvement. It employed a meta-analytic approach, incorporating data from 135 studies with a large aggregate sample size. The findings can be summarized in eight major categories.

First, three critical Self-Determination Theory (SDT) elements autonomy, competence, and relatedness—significantly and positively impact individuals' self-determination motivations. Individuals who feel autonomous tend to develop intrinsic motivation toward health-related activities that align with their values and interests. Competence is crucial as individuals strive to acquire new skills and master health-related tasks. Relatedness is reinforcing, as social connections enhance motivation by providing support, fostering a cycle that drives self-determination toward healthy behaviors (Hollembeak and Amorose, 2005; Chen and Hypnar, 2015; Mallia et al., 2019).

Second, perceived benefits, specifically perceived usefulness and enjoyment, significantly impact individuals' perceived value of engaging in health behaviors. According to the Technology Acceptance Model (TAM), these two factors enhance perceived value related to technology-enabled health behaviors (Kim et al., 2007; Yu et al., 2019; Khasawneh and Haddad, 2020).

Third, perceived sacrifices, specifically technical complexity and costs, negatively affect perceived value toward health behaviors. Technicality, reflecting the complexity of health technologies, can deter individuals from participating in health-related activities. Similarly, perceived fees can induce financial burdens, diminishing the overall perceived value and discouraging individuals from adopting healthy behaviors (Lin et al., 2012; Yu et al., 2019; Lin et al., 2020).

Fourth, self-determination motivations, as outlined in SDT, positively influence both attitudes and perceived value toward health behaviors. Drawing from the Theory of Planned Behavior (TPB), these positive attitudes can lead to the formation of behavioral intentions. Simultaneously, based on the Value-Attitude Model (VAM), self-determination motivations also enhance individuals' perceived value of health behaviors (Chen and Hypnar, 2015; Johnson et al., 2016; Carfora et al., 2022).

Fifth, individuals' attitudes toward health behaviors positively impact their perceived value, and both contribute to the likelihood of engaging in health behavior interventions. While TPB suggests that positive attitudes foster intentions to perform health behaviors, VAM argues that perceived value, influenced by attitudes, strengthens individuals' intentions to implement health behaviors (Woo and Kim, 2019; Bavaresco et al., 2020; Hasan and Suciarto, 2020).

Sixth, the intrinsic motivations shaped by autonomy, competence, and relatedness encourage individuals to engage in health behaviors. This fosters a sustainable and effective approach to both intention and implementation of health behaviors (Hagger et al., 2012; Joo et al., 2018).

Seventh, implementation intentions toward health behaviors significantly impact action planning and coping planning, and coping planning positively influences action planning, as suggested by the Health Action Process Approach (HAPA) (Schwarzer, 2008). Stronger implementation intentions lead to more effective action and coping planning, which are essential for executing health-related activities (de Bruijn et al., 2012; Caudroit et al., 2014; Hsieh et al., 2019; Zhu et al., 2022). Coping planning, in particular, helps individuals anticipate and overcome obstacles during the implementation phase, further enhancing action planning (Scholz et al., 2008; Pakpour et al., 2011; Godinho et al., 2014; Hsieh et al., 2019).

Finally, implementation intentions, action planning, and coping planning all significantly and positively impact health behaviors, leading to overall health improvement. According to HAPA, implementation intentions are a prerequisite for health behavior changes. Action planning provides a clear roadmap, specifying when, where, and how to execute health behaviors while coping planning designs strategies to overcome barriers. Together, they facilitate health behavior execution and mitigate potential setbacks, promoting long-term health improvement (Hagger et al., 2012; Allan et al., 2013; Lena et al., 2019; Rogowska et al., 2021; Zhu et al., 2022).

### 5.1 Academic implications

In the context of health behaviors and health improvements, this study is the first to integrate the Theory of Planned Behavior (TPB), Self-Determination Theory (SDT), Technology Acceptance Model (TAM), Value-Attitude Model (VAM), and Health Action Process Approach (HAPA) into a comprehensive research framework. A meta-analysis was conducted to examine the hypotheses within this framework by combining the findings of 135 research for a total sample size of 53,242. This allowed for estimating the effect size and variability across numerous investigations. The study's conclusions, divided into eight major themes as shown above, offer insightful information about the variables influencing decisions made concerning one's health. These findings have various academic ramifications.

First, a deep knowledge of how internal motives impact decisions relating to health is provided by recognizing autonomy, competence, and relatedness as fundamental aspects of SDT. This discovery is essential for scholars and investigators examining the psychological foundations of health-related behaviors. The research on health behavior change processes gains depth when it is realized how these SDT components influence people's motivation to engage in health-related activities.

Second, applying TAM principles emphasizes how important it is for people to understand the importance of perceived benefits—specifically perceived utility and enjoyment—in determining how important they think it is to adopt healthy behaviors. This discovery advances our knowledge of the psychological factors that influence technology-enabled health intentions, especially in light of the growing use of digital health technologies. Concurrently, admitting the detrimental effects of perceived costs and sacrifices, including perceived technical difficulty, emphasizes how critical it is to remove obstacles to participation. Researchers should investigate further how lowering these obstacles can improve the perceived value and promote the uptake of health innovations.

Third, consistent with TAM, VAM, and TPB, the favorable impacts of self-determination motivations on attitudes and perceived value deepen our understanding of the complex interplay between motivational variables and attitudes related to health. Scholars examining the psychological mechanisms influencing individual decision-making in health-related contexts need a deeper grasp of how incentives impact attitudes.

Fourth, the study highlights the interaction among attitudes, perceived value, and implementation intentions, reaffirming that cultivating a good attitude is a prerequisite for motivating behavioral intentions and consequent modifications in health-related behavior. This information can help researchers better understand how attitude formation affects health decisions and illuminate the processes that result in action.

Fifth, the emphasis on SDT elements like relatedness, competence, and autonomy provides a comprehensive understanding of the goals and practices of sustainable health behavior. These elements are essential in guaranteeing that people's reasons for practicing healthy habits are self-determined and long-lasting. This technique offers a solid theoretical foundation for researchers studying long-term health behavior change to investigate the relationship between intrinsic motivation and long-term health outcomes.

The research framework's incorporation of HAPA components highlights the significance of implementation intention, action planning, and coping planning in bridging the gap between behavior change's motivational and volitional stages. This combination gives academics a thorough grasp of how people turn aspirations about general health into specific actions. Through analyzing the mechanisms behind action and coping planning, researchers can gain a deeper understanding of the valuable tactics that support the effective implementation of healthy behaviors. Acknowledging the beneficial effects of coping plans, action plans, and implementation intents on health behaviors provides researchers with a more detailed understanding of how these elements support health enhancement. This knowledge enhances the field by providing a more comprehensive understanding of the mechanisms behind long-term wellbeing and successful health behavior modifications.

This study offers several practical advantages besides its scholarly achievements. The findings underscore the importance of autonomy, competence, and relatedness in fostering health habits. This information may aid policymakers in formulating initiatives that promote sustainable health practices and empower individuals. Understanding these psychological variables may help develop policies that promote ongoing health participation, particularly in health maintenance and prevention. The study emphasizes perceived advantages (utility and pleasure) and perceived drawbacks (specifically technical complexity) in adopting technology-enabled health therapies. Empirical results indicate that digital health developers may enhance user engagement by addressing these characteristics, making health technology more user-friendly and attractive, hence promoting better habits.

Furthermore, the research provides insights into how motivational factors influence health-related attitudes and intentions, offering valuable guidance for designing personalized health interventions. By targeting these motivational drivers, practitioners can enhance the effectiveness of interventions and improve individuals' engagement with health-promoting behaviors. The inclusion of HAPA in the framework emphasizes the importance of implementation intentions, action planning, and coping strategies in facilitating the transition from motivation to sustained behavior change, offering a solid foundation for behavioral interventions that support long-term health improvements.

In conclusion, the synthesis of multiple theoretical models provides a deeper academic understanding of health behavior and actionable insights that can improve health interventions, policies, and technologies. These practical contributions guide future research and applications to promote long-term health behavior changes.

### 5.2 Managerial implications

For managers, marketers, and top management teams, the conclusions taken from this research results offer strategic insights from decision-making.

First, the influence of SDT elements on individuals' self-determination motivations can lead managers to generate solutions that connect with individuals' psychological needs, supporting sustained involvement in health-related activities. Second, recognizing the positive impact of perceived usefulness and enjoyment and the adverse effects of technicality and perceived fees on perceived value toward health behavior can provide marketers with variable insight for crafting communication strategies to promote individuals' perceived value toward health behavior. Firms and managers should endeavor to create health-related activities that are more beneficial and provide a fun environment with simple and acceptable technology and lower fees. Managers should use these findings to build workplace initiatives on wellness programs that line with individuals' inherent motivations. Third, since encouraging good attitudes and perceived value becomes the primary purpose of enterprises, managers should establish communication techniques that can reinforce positive attitudes and highlight the perceived value of health-related activities. Fourth, adopting a holistic perspective emphasizing the interdependence of autonomy, competence, and relatedness provides managers with a roadmap for implementing comprehensive health solutions. This insight can lead organizations' top measurement teams in establishing policies that promote individuals' holistic wellbeing. Fifth, managers should combine HAPA principles into a strategic move to emphasize the importance of implementation intentions, action planning, and copying planning to support individuals in overcoming hurdles during health behavior and improvement. Finally, evaluating the influence of implementation intention, action planning, and coping planning on health behavior might inform managerial decisions related to resource allocation and program development. Providing practical tools and resources that enable these action and coping planning processes can boost health programs' effectiveness and long-term success.

### 5.3 Limitations and future research directions

This study utilized meta-analysis to test the research hypotheses. The strengths of meta-analysis lie in its ability to combine data from multiple studies, thereby increasing statistical power and providing more robust conclusions. It allows for assessing the overall effect size of each hypothesis, exploring potential sources of heterogeneity, and revealing patterns, trends, and effects that may not be apparent in individual studies. However, meta-analyses have several limitations, including dependence on the quality of the included studies, potential publication bias, the risks associated with combining studies using different methodologies, and the availability of limited data from prior research.

Specifically, although many datasets have been included in this study, their data sources and collection methods may vary significantly. Additionally, this meta-analysis uses correlations (r) rather than estimates (β) to assess relationships, limiting the ability to infer causal relationships among research constructs. The directional influence paths of each hypothesis were derived from theory rather than the data itself. Consequently, causal inferences about the relationships between variables should be interpreted cautiously.

Second, although this study has successfully integrated key dimensions from SDT, TAM, VAM, TPB, and HAPA, some critical health behavior and improvement dimensions might be absent. For instance, the influence of action control, experiential perception, and emotional arousal could provide further insights into understanding health behavior change (Zhang et al., 2019). Future research could incorporate these dimensions to develop a more comprehensive framework that captures the full spectrum of factors influencing health behaviors.

Third, moderating variables, such as age, gender, educational level, cultural/geographical region, and study periods, can be used to explain the variance of influences between different groups. Additional moderating variables may enhance the current research model. Prior studies have suggested that individuals' implementation intentions may act as moderators, strengthening the impact of self-determination motivations on health behaviors and improvement. Similarly, action planning could moderate the relationship between implementation intentions and health behaviors, while perceived risk might moderate the impact of self-efficacy on health behavior (Zhang et al., 2019). Future research should consider incorporating these moderators into the model to provide a more nuanced understanding of the factors influencing health behavior and improvement. As more studies explore moderating effects using continuous variables, synthesizing moderator effects through meta-analysis has become increasingly feasible. Future research could build upon this foundation by examining the role of continuous variables as moderators and their interaction effects in the context of health behavior.

Finally, while this study contributes significantly to the existing body of knowledge by proposing an integrative framework that combines multiple theories and perspectives on health behavior and improvement, it does not claim to present the definitive model for understanding health behavior. Several questions remain open for future investigation: (1) Does this model explain the majority of variance in health behavior outcomes? (2) Does it provide the most accurate insights into the causal mechanisms of health behavior and improvement? (3) Is this model the most effective for designing interventions to promote health behavior change? Future studies could challenge the current model by addressing these questions and refining the framework to further advance theoretical and practical understanding (Schwarzer and Luszczynska, 2008).

Finally, while providing a solid basis for understanding health habits and improving health, this study also leaves room for additional research and development. Future research should incorporate absentee components, investigate more moderating factors, and correct the flaws found to produce a more comprehensive and trustworthy model for health behavior modification.

## Data Availability

The original contributions presented in the study are included in the article/supplementary material, further inquiries can be directed to the corresponding author.
